# Pre-vascularized enhances therapeutic effects of human bone marrow-derived mesenchymal stem cell sheets in Buccal Mucosa wound

**DOI:** 10.1186/s12903-026-08189-7

**Published:** 2026-04-07

**Authors:** Yuhang Zhang, Yingjie Zhang, Qian Dong, Zhaoqiang Zhang, Zhe Ji, Yun Deng, Yu Chen

**Affiliations:** 1 Department of Stomatology, The First Affiliated Hospital of Guangzhou Medical University, Guangzhou, Guangdong 510120 China; 2 Department of Stomatology, The First Affiliated Hospital of Sun Yat-sen University, Guangzhou, Guangdong 510080 China; 3 Center for Information Technology and Statistics, The First Affiliated Hospital of Sun Yat-sen University, Guangzhou, Guangdong 510080 China; 4 Stomatological Hospital, School of Stomatology, Southern Medical University, Guangzhou, Guangdong 510280 China; 5 Hospital of Stomatology, Guanghua School of Stomatology, Guangdong Provincial Key Laboratory of Stomatology, Sun Yat-sen University, Guangzhou, Guangdong 510055 China

**Keywords:** Mucosal graft, Human mesenchymal stem cell, Prevascularized cell sheet, Angiogenic growth factors

## Abstract

**Background:**

Lesions of the buccal mucosa impair normal oral function and are often accompanied by pain. While biomaterials has demonstrated potential in promoting wound healing, its efficacy may be limited by insufficient early vascularization. This study was conducted to investigate the oral mucosa repair efficacy and regenerative potential of human bone marrow-derived mesenchymal stem cell sheets (HCS) and pre-vascularized HCS (PHCS).

**Method:**

A total of 105 rats were randomly assigned to one of three groups based on the type of graft applied to the wound bed: (1) control group: biomembranes alone, (2) HCS group: biomembranes combined with HCS, and (3) PHCS group: biomembranes combined with PHCS. HCS and PHCS were respectively combined with biomembranes and applied to full-thickness buccal mucosal wounds in rats. The wounds were observed at days 3, 5, 7, 10, and 13 post-operation and Hematoxylin and eosin staining and Masson’s Trichrome staining were conducted.

**Results:**

Compared with the biomembrane-only control group, both HCS and PHCS treatments significantly reduced mucosal contraction and improved wound appearance. The PHCS group exhibited the least bleeding and necrosis, minimal inflammatory cell infiltration, and the highest level of early-stage revascularization. This led to the establishment of a robust microcirculation, supporting graft survival and tissue regeneration. Furthermore, PHCS grafts resulted in the thinnest epithelial layer, indicative of more favorable healing outcomes.

**Conclusion:**

The study suggest that the combination of biomembranes and PHCS implantation further improve regenerative outcomes by providing a richer source of angiogenic factors and preformed microvascular structures, therefore presents a promising therapeutic strategy for the regeneration of full-thickness buccal mucosal defects.

**Clinical trial number:**

Not applicable.

## Introduction

Extensive oral mucosal injuries can be classified as either primary or secondary in origin and are typically associated with tumors, trauma, mucosal diseases, and preprosthetic surgical interventions. These lesions frequently extend into the submucosa, resulting in full-thickness mucosal damage, characterized by complete destruction of both the epithelial layer and the underlying connective tissue, including partial or total loss of the submucosa. In severe cases, the damage may reach the muscular layer or even deeper anatomical structures. Such extensive tissue compromise leads to a significant loss of essential mucosal functions, including barrier protection, lubrication, and immunological defense. Clinically, this often presents with pain, bleeding, an elevated risk of infection, and impaired oral function [1]. Due to the absence of native progenitor cells and structural scaffolds, natural healing is typically slow and often requires surgical intervention (e.g. primary closure, mucosal graft, split-thickness skin grafts (STSGs), and microvascular pedicle grafts) [2] or the application of biomaterials (e.g. acellular matrices, collagen membranes, hyaluronic acid hydrogels, hydrogel adhesives) to facilitate effective tissue regeneration [3–6].

For full-thickness mucosal defects, surgical approaches remain invasive, and the application of autologous grafts is often constrained by limited donor tissue availability. While cellular or acellular biomaterial-based therapies presents a promising alternative to address these limitations [2], the clinical efficacy of these materials remains suboptimal, primarily due to their slow degradation rates, insufficient early vascularization, and restricted regenerative capacity in large-scale oral mucosal reconstruction [6].

As vascular regeneration is crucial for successful mucosal healing [7], promoting angiogenesis in the wound bed has become a central therapeutic goal. Recent studies have demonstrated that human bone marrow-derived mesenchymal stem cell (hBMSC) sheets can significantly accelerate wound healing. These cell sheets are cultivated under hyperconfluent conditions, allowing for extensive cell-cell interaction and endogenous extracellular matrix production, resulting in a cohesive sheet-like structure [8]. In a recent study, hBMSCs have been successfully applied to repair various tissues including cartilage, bone, and periodontal structures, and they thrive in hypoxic microenvironments, such as those found in bone marrow [9]. Under these conditions, hBMSCs secrete trophic factors that promote angiogenesis and function as pericytes to stabilize newly formed endothelial capillary networks [10].

Multiple studies have reported that hypoxic preconditioning enhances the angiogenic potential and vascular integration capacity of hBMSCs [10, 11]. Moreover, co-culturing hBMSCs with human umbilical vein endothelial cells (HUVECs) under hypoxic conditions facilitates the formation of pre-capillary networks within three-dimensional (3D) scaffold constructs [10–12]. These pre-vascularized hBMSC cell sheets (PHCS) exhibit significant potential in promoting early vascularization and integration of grafted tissues [10].

The present study aims to evaluate the regenerative potential of hBMSC cell sheets (HCS) and PHCS in enhancing tissue repair by delivering angiogenic factors and pre-formed microvascular structures, thereby improving graft survival, integration, and functional recovery.

## Materials and methods

### Ethics statement

All experimental protocols in this study, including the animal experiments and the use of human-derived cells for transplantation into animal models, were reviewed and approved by the Ethics Committee and the Institutional Animal Care and Use Committee (IACUC) of the First Affiliated Hospital of Sun Yat-sen University (Approval No. [2017]273).

This study was conducted in accordance with the ethical principles of the Declaration of Helsinki. The approval covered the use of commercially obtained, de-identified human cells in vivo for tissue engineering and regenerative research purposes. The hBMSCs were obtained from Texas A&M University Health Sciences Center, and HUVECs were purchased from Lonza©, Walkersville, MD. According to the providers, informed consent was obtained from all donors, and the procurement and research use of these cells were approved by the corresponding Institutional Review Board (IRB) or ethics committee. All cell samples were de-identified prior to distribution, and no personally identifiable information was accessible to the authors.

The use of human-derived cells for in vivo transplantation experiments was explicitly covered by the approved experimental protocol. This study did not involve the generation of germline chimeras, neural tissue integration, or transplantation into reproductive organs. All experiments involving human cells in live animal models were conducted in compliance with internationally recognized ethical and biosafety standards, including International Society for Stem Cell Research (ISSCR) guidelines [13–15].

All animal procedures, including surgical transplantation, postoperative monitoring, and euthanasia, were conducted in strict accordance with national regulations ARRIVE guidelines, with efforts made to minimize animal suffering and reduce animal use.

### Preparation of hBMSC cell sheet (HCS) and pre-vascularized hBMSC cell sheets (PHCS)

**Fig. 1:**
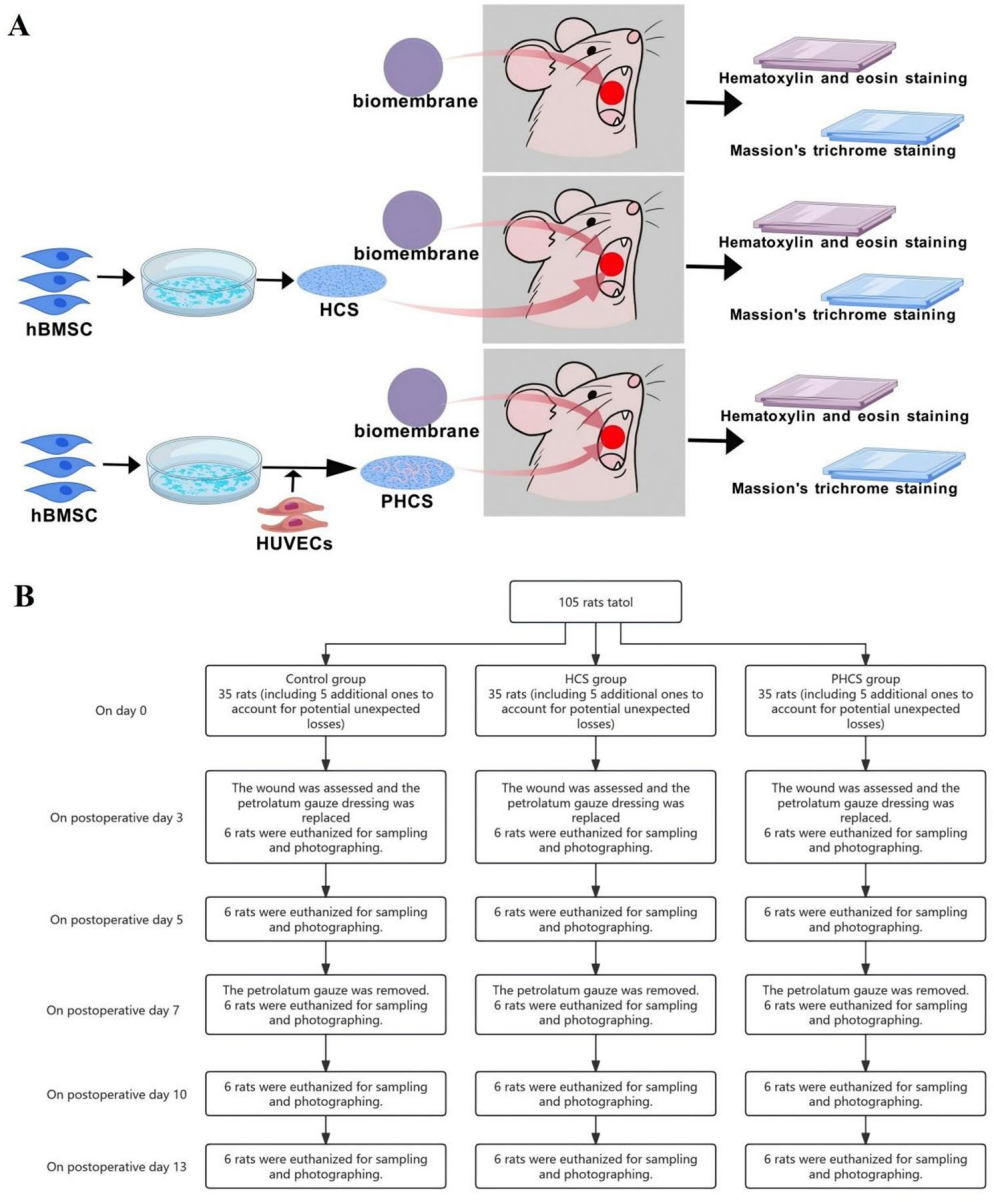
Schematic illustration of the experimental design. **A** Control group (top): A biomembrane was directly applied to the oral mucosal defect in rats, followed by histological evaluation using hematoxylin and eosin (H&E) staining and Masson’s trichrome staining; HCS group (middle): Human bone marrow mesenchymal stem cell (hBMSC)-derived cell sheets (HCS) were transplanted into the defect area and covered with a biomembrane, followed by H&E and Masson’s staining; PHCS group (bottom): Co-cultured hBMSC and human umbilical vein endothelial cell (HUVEC)-derived prevascularized cell sheets (PHCS) were transplanted into the defect area and covered with a biomembrane, followed by H&E and Masson’s staining. **B** A total of 105 rats were randomly assigned into three groups: Control, HCS, and PHCS (*n* = 35 per group). On day 3, the petrolatum gauze was removed to inspect the wound and replaced with a new dressing. On day 7, the gauze was completely removed. At each time point (days 3, 5, 7, 10, and 13), six rats per group were euthanized for specimen collection and wound photography, which was used for size measurement

To optimize the structural integrity of hBMSC cell sheets and promote the formation of microvascular networks, culture conditions for both HCS and PHCS were refined (Fig. 1). Briefly, hBMSCs (passages 3–5, obtained from Texas A&M University Health Sciences Center) were seeded onto collagen I-coated (20 µg/mL; BD Biosciences, San Jose, CA) glass coverslips at a density of 10,000 cells/cm² [11]. The cells were cultured under hypoxic conditions (2% O_2_) for four weeks in *α*-minimum essential medium (*α*-MEM) supplemented with 20% fetal bovine serum, 1% L-glutamine, and 1% penicillin/streptomycin (Life Technologies, Rockville, MD) to form HCS [10].

To generate PHCS, HUVECs (Lonza, Walkersville, MD) were seeded onto the surface of the hBMSC cell sheets at a density of 20,000 cells/cm². Co-cultures were maintained under normoxic conditions (20% O_2_) for one week to allow the formation of microvascular networks within the three-dimensional scaffold [10].

### Animals

Sprague Dawley (SD) rats, weighing between 180 and 200 g, were obtained from the Experimental Animal Center of Sun Yat-sen University. A total of 105 rats were randomly assigned to one of three groups (*n* = 35 per group) based on the type of graft applied to the wound bed [16]: (1) control group: biomembranes alone, (2) HCS group: biomembranes combined with HCS, and (3) PHCS group: biomembranes combined with PHCS (Fig. 1).

### Full thickness excision wound creation and graft transplantation

All rats were anesthetized via intraperitoneal injection of pentobarbital sodium (30–50 mg/kg) prior to surgery and euthanasia was performed by intraperitoneal injection of pentobarbital sodium (Nembutal^®^, USA) at a dose of 200 mg/kg to induce deep anesthesia after experiments. The perioral region was aseptically prepared through sequential cleansing with povidone-iodine surgical scrub (Betadine Veterinary) followed by 75% ethanol. Oral mucosal surfaces were disinfected with 0.12% chlorhexidine gluconate and subsequently irrigated with sterile saline. Under aseptic conditions, a standardized full-thickness circular wound (8 mm in diameter) was surgically created in the buccal mucosa using iris scissors.

Following wound creation, three cell sheets were applied to each wound site, over which a resorbable collagen membrane (SureDerm^®^, Korea) was placed. In the control group, the biomembrane was applied without any underlying cell sheet. The grafts and surrounding wound margins were secured using interrupted sutures. To ensure stable graft adherence and minimize mechanical disruption, a petrolatum gauze dressing was applied and sutured with 7 − 0 black braided silk (Mersilk, Ethicon, Inc., USA) to provide uniform pressure and prevent scratching. The gauze dressings were changed on postoperative day 3 and removed on day 7, while the membrane was left undisturbed. Rats were euthanized at days 3, 5, 7, 10, and 13 post-operation for specimen collection and wound photography [16–18].

Wound contraction was quantitatively assessed using gravitational planimetry. The contraction rate was calculated as the percentage reduction in wound area relative to the original wound size using the following formula [19]:

$$\begin{array}{ll}\mathrm{R}\mathrm{e}\mathrm{l}\mathrm{a}\mathrm{t}\mathrm{i}\mathrm{v}\mathrm{e}\:\mathrm{m}\mathrm{u}\mathrm{c}\mathrm{o}\mathrm{s}\mathrm{a}\:\mathrm{g}\mathrm{r}\mathrm{a}\mathrm{f}\mathrm{t}\:\mathrm{s}\mathrm{i}\mathrm{z}\mathrm{e}\:\left(\%\right)\\=\frac{\mathrm{A}\mathrm{r}\mathrm{e}\mathrm{a}\:\mathrm{o}\mathrm{f}\:\mathrm{R}\mathrm{e}\mathrm{m}\mathrm{a}\mathrm{i}\mathrm{n}\mathrm{i}\mathrm{n}\mathrm{g}\:\mathrm{M}\mathrm{u}\mathrm{c}\mathrm{o}\mathrm{s}\mathrm{a}\:\mathrm{G}\mathrm{r}\mathrm{a}\mathrm{f}\mathrm{t}}{\mathrm{A}\mathrm{r}\mathrm{e}\mathrm{a}\:\mathrm{o}\mathrm{f}\:\mathrm{O}\mathrm{r}\mathrm{i}\mathrm{g}\mathrm{i}\mathrm{n}\mathrm{a}\mathrm{l}\:\mathrm{W}\mathrm{o}\mathrm{u}\mathrm{n}\mathrm{d}}\times\:100\:\left(\%\right)\end{array}$$

### Tissue collection

Rats were euthanized at predetermined time points, specifically at days 3, 5, 7, 10, and 13 post-operation, with six rats per group for each time point. The grafts and surrounding tissues were harvested and sectioned along the centerline into two equal parts. All samples were subsequently fixed in 10% formalin at room temperature, followed by dehydration through a graded series of ethanol solutions, and then embedded in paraffin.

### Pathology analysis: staining, imaging, and analysis

After dewaxation and rehydration, tissue sections with 5 μm thickness were prepared for staining. Hematoxylin and eosin (HE) staining and Masson’s Trichrome staining were conducted according to the manufacturer’s (Sigma) standardized protocols. H&E staining was utilized to assess the microstructural architecture and cellular morphology within the grafts. Masson’s Trichrome staining was employed to evaluate collagen fiber deposition and maturation.

Measurements of epidermal thickness, microvessel density, gland count, and cell number counting in high-power field (HPF) were performed with Image J software [19–21]. Microvessel area was defined as the total area of all vessel segments within a high-power field (HPF) and quantified based on previously described image analysis methods [19]:

$$\begin{array}{ll}\mathrm{V}\mathrm{e}\mathrm{s}\mathrm{s}\mathrm{e}\mathrm{l}\:\mathrm{i}\mathrm{n}\mathrm{d}\mathrm{e}\mathrm{x}\:\left(\%\right)\\=\frac{\mathrm{A}\mathrm{r}\mathrm{e}\mathrm{a}\:\mathrm{o}\mathrm{f}\:\mathrm{s}\mathrm{u}\mathrm{m}\:\mathrm{o}\mathrm{f}\:\mathrm{a}\mathrm{l}\mathrm{l}\:\mathrm{v}\mathrm{e}\mathrm{s}\mathrm{s}\mathrm{e}\mathrm{l}\:\mathrm{s}\mathrm{e}\mathrm{g}\mathrm{m}\mathrm{e}\mathrm{n}\mathrm{t}\mathrm{s}}{\mathrm{A}\mathrm{r}\mathrm{e}\mathrm{a}\:\mathrm{o}\mathrm{f}\:\mathrm{H}\mathrm{P}\mathrm{F}}\times\:100\:\left(\%\right)\end{array}$$$$$$$$$$$$$$$$\begin{array}{ll}\mathrm{V}\mathrm{e}\mathrm{s}\mathrm{s}\mathrm{e}\mathrm{l}\:\mathrm{i}\mathrm{n}\mathrm{d}\mathrm{e}\mathrm{x}\:\left(\%\right)\\=\frac{\mathrm{A}\mathrm{r}\mathrm{e}\mathrm{a}\:\mathrm{o}\mathrm{f}\:\mathrm{s}\mathrm{u}\mathrm{m}\:\mathrm{o}\mathrm{f}\:\mathrm{a}\mathrm{l}\mathrm{l}\:\mathrm{v}\mathrm{e}\mathrm{s}\mathrm{s}\mathrm{e}\mathrm{l}\:\mathrm{s}\mathrm{e}\mathrm{g}\mathrm{m}\mathrm{e}\mathrm{n}\mathrm{t}\mathrm{s}}{\mathrm{A}\mathrm{r}\mathrm{e}\mathrm{a}\:\mathrm{o}\mathrm{f}\:\mathrm{H}\mathrm{P}\mathrm{F}}\times\:100\:\left(\%\right)\end{array}$$

Collagen deposition was semi-quantitatively assessed using Masson’s Trichrome-stained histological sections. Digital images were captured under identical microscopy settings and analyzed using ImageJ software. The blue-stained area corresponding to collagen fibers was selected using a consistent color threshold, and the collagen deposition was expressed as the percentage of blue-stained area relative to the total tissue area within the region of interest. The results were reported as collagen area fraction (%) [22].

For each group and time point, stained sections were visualized using an Olympus IX71 microscope, and three random fields per section were selected for quantitative assessment.

### Statistics/data analysis

The results are presented as mean±standard deviation. Statistical comparisons between conditions were performed using one-way analysis of variance (ANOVA) or Student’s t-test, as appropriate. A p-value of less than 0.05 was considered statistically significant.

## Result

### Mucosa grafts contraction

**Fig. 2:**
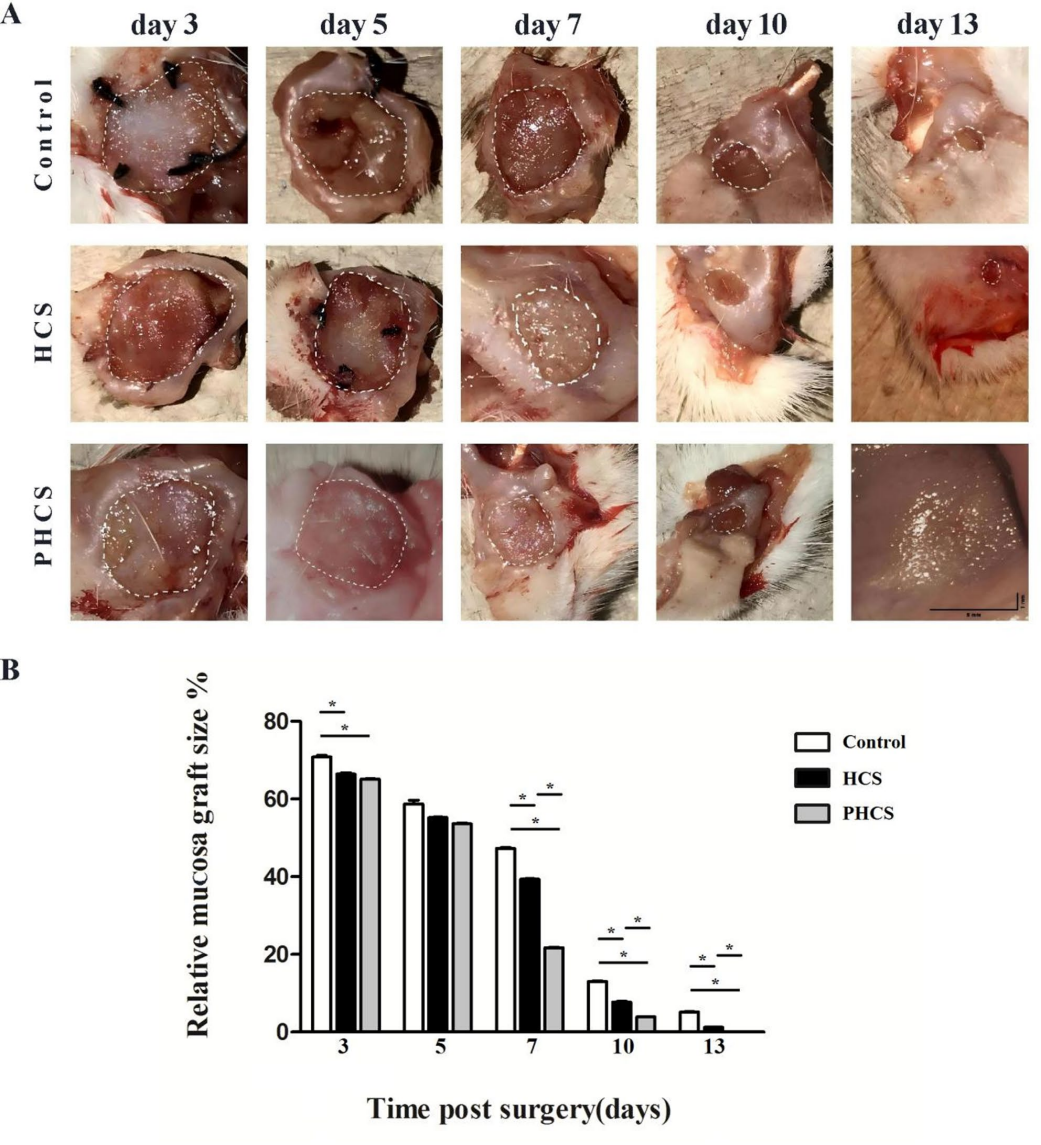
Macroscopic evaluation of mucosal wound healing. **A** Representative macroscopic images of mucosal defects in the Control, HCS, and PHCS groups on postoperative days 3, 5, 7, 10, and 13. Progressive wound closure was observed in all groups, with the PHCS group exhibiting accelerated healing. Scale bar = 50 mm. **B** Quantitative analysis of relative mucosal graft size (%) at different time points post-surgery. The PHCS group showed significantly faster wound closure compared to the HCS and Control groups. *p < 0.05

The graft margins in all three groups exhibited a reddish appearance with localized ulceration in the non-healed regions (Fig. 2). On postoperative day 3, the degree of graft contraction was comparable between the HCS and PHCS groups (*p* > 0.05), while the control group displayed a slightly larger graft area compared to the other two groups (*p* < 0.05). By day 5, no significant differences in graft contraction were observed among the three groups (*p* > 0.05). However, on day 7, a significant reduction in graft area was noted in the PHCS group (*p* < 0.05). By day 10, the PHCS group had nearly achieved complete healing, whereas the control group retained a significantly larger residual graft area (13.00 ± 0.24%) compared to the HCS group (7.27 ± 0.25%, *p* < 0.05). By day 13, the HCS group also exhibited near-complete healing, although a measurable amount of residual graft tissue remained in the control group (5.20 ± 0.19%, *p* < 0.05).

### Epithelial thickness of the oral mucosa

**Fig. 3:**
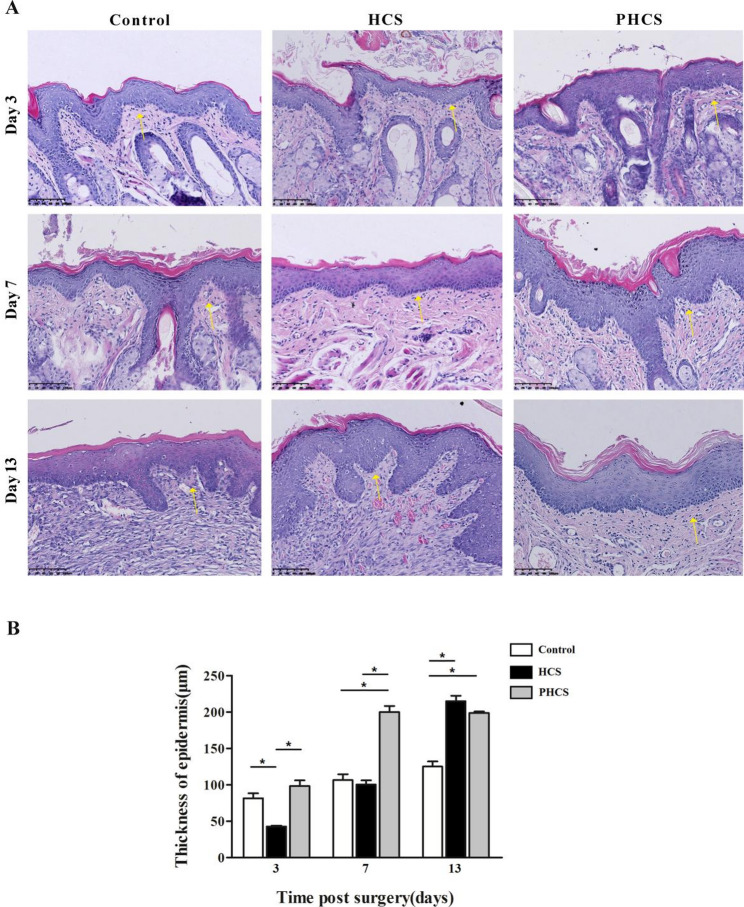
Histological evaluation of epithelial regeneration. **A** Representative H&E-stained sections of epithelial tissues in the Control, HCS, and PHCS groups at postoperative days 3, 7, and 13. On day 3, the HCS group displayed the thinnest epithelium, while the PHCS group exhibited greater epithelial cell density and stratification compared to the Control. By day 7, epithelial thickness nearly doubled in both the HCS and PHCS groups, with the PHCS group showing significantly greater thickness. By day 13, epithelial thickness in the HCS group increased to nearly fivefold of the baseline, and both HCS and PHCS groups demonstrated significantly greater epithelial thickness than the Control, with the PHCS group exhibiting more orderly epithelial organization. Scale bar = 100 μm. **B** Quantitative analysis of epithelial thickness at different time points. *p < 0.05

On postoperative day 3, the HCS group exhibited the thinnest epithelial layer. Although there was no significant difference in epithelial thickness between the PHCS and control groups (*p* > 0.05), the PHCS group demonstrated a greater number and density of epithelial cell layers compared to the control group (Fig. 3). By day 7, epithelial thickness in both the HCS and PHCS groups had approximately doubled relative to day 3. At this time point, the HCS group’s epithelial thickness was comparable to that of the control group (*p* > 0.05), whereas the PHCS group showed significantly greater epithelial thickness than both the HCS and control groups (*p* < 0.05). Additionally, the PHCS group exhibited higher cellular density and stratification, although the cell arrangement remained disordered. By day 13, epithelial thickness in the HCS group continued to increase, reaching approximately five times the initial measurement. Compared with the control group (125.10 ± 12.20 μm), both the HCS (215.00 ± 13.05 μm) and PHCS (198.70 ± 3.60 μm) groups exhibited significantly greater epithelial thickness (*p* < 0.05). While there was no significant difference in epithelial thickness between the PHCS and HCS groups (*p* > 0.05), the epithelial cell arrangement in the PHCS group appeared more orderly.

### Comparative histological evaluation of inflammatory response, collagen deposition, and glandular regeneration

**Fig. 4:**
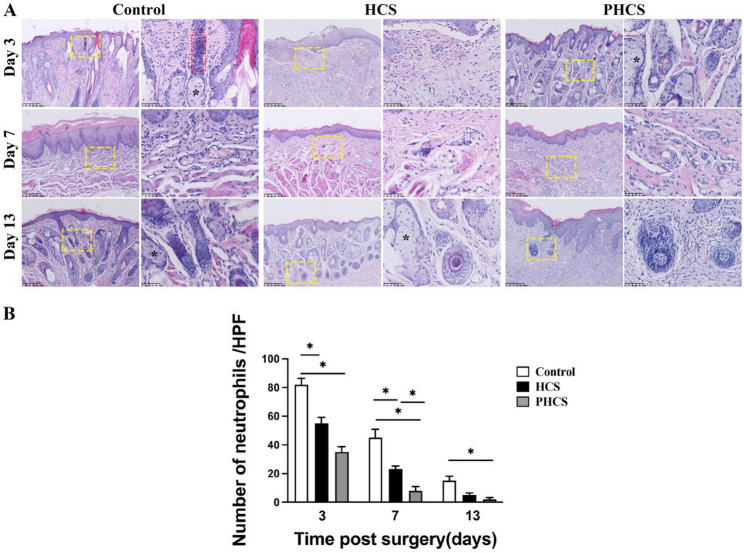
Comparative Histological Evaluation of Inflammatory Response, Collagen Deposition, and Glandular Regeneration. **A** Representative H&E-stained sections of wound tissues in the Control, HCS, and PHCS groups at postoperative days 3, 7, and 13. Yellow boxes indicate magnified regions. HE staining of epithelial section showing glands (black stars), and neutrophils (red dashed rectangles). On day 3, abundant neutrophil infiltration was observed in the Control group, moderate infiltration in the HCS group, and mild infiltration in the PHCS group. By day 7, neutrophil infiltration decreased in all groups, with the lowest level in the PHCS group. By day 13, only a few neutrophils remained, particularly in the PHCS group. Scale bar = 100 μm. **B** Quantitative analysis of neutrophil counts per high-power field (HPF) at different time points. *p < 0.05

The control group exhibited pronounced histological signs of inflammation, including extensive hemorrhage, marked neutrophilic infiltration, and poorly developed glandular structures with disrupted architecture. Collagen deposition was minimal, and the collagen fibers appeared disorganized (Fig. 4).

In contrast, the HCS group showed moderate histological improvement across several parameters. Compared with the control group, hemorrhage and neutrophilic infiltration were reduced, and collagen fibers were more uniformly arranged. Tissue architecture was moderately improved, indicating enhanced healing. However, collagen deposition remained suboptimal, and glandular structure formation was still incomplete.

The most favorable histological outcomes were observed in the PHCS group, which demonstrated significant improvements across all evaluated parameters. Specifically, the PHCS group exhibited: (1) minimal hemorrhage and neutrophil infiltration; (2) a substantial increase in the number of glandular structures with well-preserved architectural integrity; and (3) the highest level of collagen deposition, characterized histologically by densely distributed and well-organized collagen fibers. Notably, by postoperative day 7, inflammatory responses had decreased across all grafts, with prominent collagen fiber organization and neovascularization emerging as the dominant histological features. By day 13, the PHCS group displayed highly organized collagen alignment and complete epithelial differentiation (Fig. 6A).

### Vascularization of mucosal grafts

**Fig. 5:**
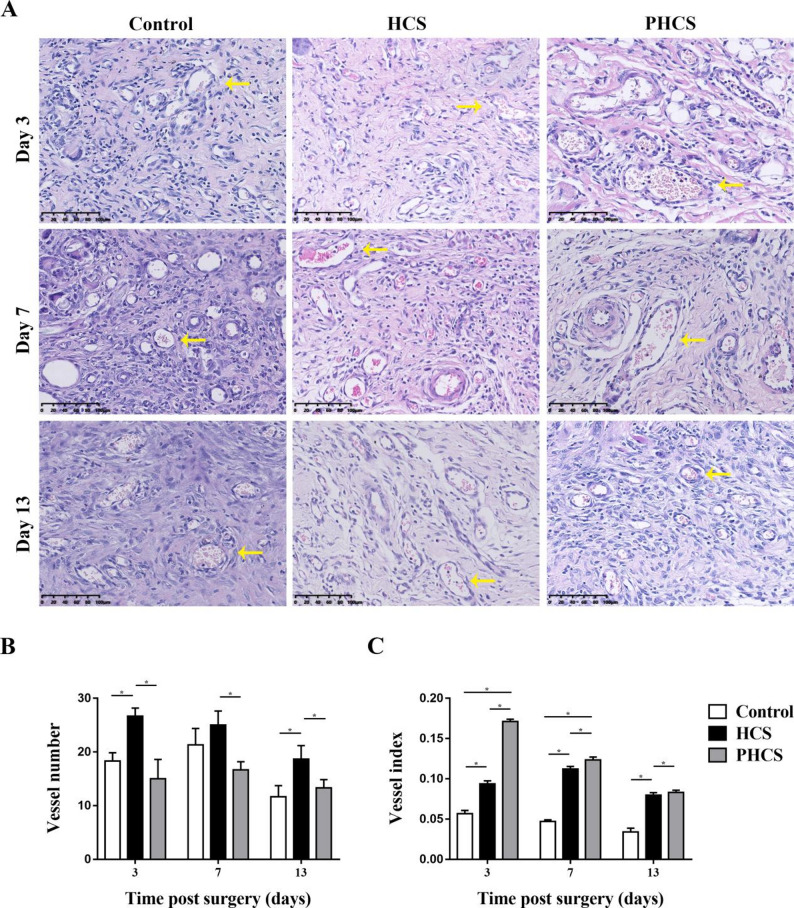
Histological evaluation of angiogenesis. **A** Representative H&E-stained sections of wound tissues in the Control, HCS, and PHCS groups at postoperative days 3, 7, and 13. Newly formed vessels are indicated by yellow arrows. On day 3, both HCS and PHCS groups exhibited more abundant neovascularization compared with the Control. By day 7, vessel formation peaked in HCS and PHCS groups, with the PHCS group showing more mature vessel structures. On day 13, vessel numbers declined in all groups, while PHCS maintained relatively higher vessel density. Scale bar = 100 μm. **B **Quantification of microvessels at day 3, 7, and 10. *p < 0.05. **C** The total area of the microvessels in the grafts at day 3, 7, and 10. *p < 0.05

HE staining was used to evaluate the morphology, quantity, and area index of microvessels within the grafted mucosal tissues (Fig. 5A-C). Microvessels were clearly observed throughout the grafts, and red blood cells were also evident. On postoperative day 3, the PHCS group exhibited significantly fewer microvessels compared to both the HCS and control groups (PHCS vs. HCS: 15.00 ± 3.60/HPF vs. 26.67 ± 1.53/HPF, *p* < 0.05; control vs. HCS: 18.33 ± 1.53/HPF vs. 26.67 ± 1.53/HPF, *p* < 0.05; control vs. PHCS: 18.33 ± 1.53/HPF vs. 15.00 ± 3.60/HPF, *p* > 0.05). Interestingly, despite the lower vessel number, the PHCS group showed a significantly higher vascular area index (17.10 ± 0.31%) compared to both the control (5.71 ± 0.21%) and HCS (9.42 ± 0.42%) groups (*p* < 0.05). By day 7, only the HCS group demonstrated a slight increase in vascular area (11.22 ± 0.37%, *p* < 0.05). From days 7 to 13, both the number and area of blood vessels declined gradually in all groups. In summary, the PHCS group exhibited a lower microvessel number but a larger vascular area throughout the healing process.

### Collagen deposition and maturation

**Fig. 6:**
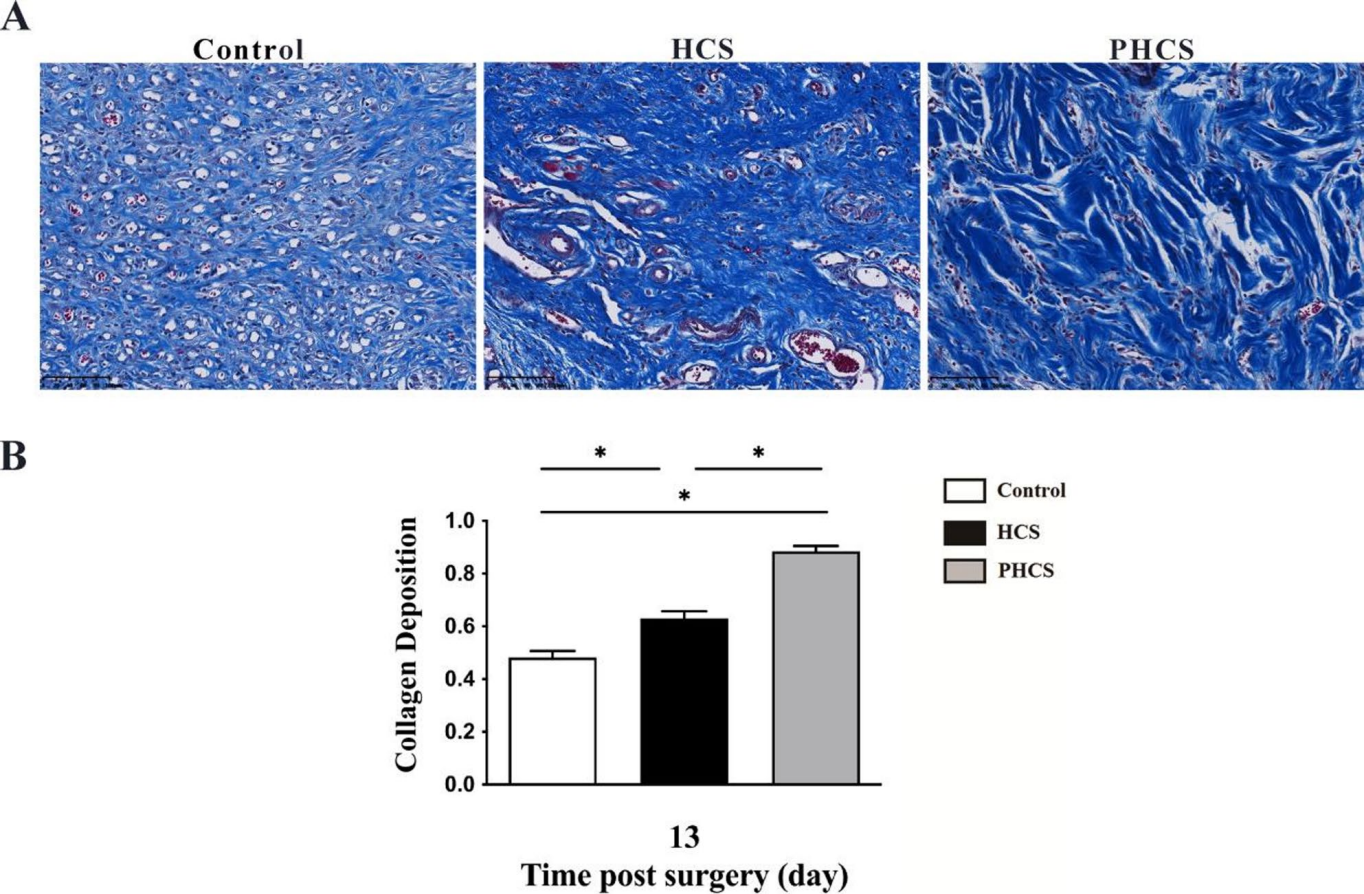
Collagen deposition at day 13 post-operation. **A** Representative Masson’s trichrome-stained sections of wound tissues in the Control, HCS, and PHCS groups at day 13. Collagen fibers are stained blue. Both HCS and PHCS groups exhibited markedly increased collagen deposition and organization compared to the Control. Scale bar = 100 μm. **B** Semi-quantitative analysis of collagen deposition. Both HCS and PHCS groups showed significantly higher collagen deposition compared to the Control, with the PHCS group exhibiting the greatest increase. *p < 0.05

Semi-quantitative image analysis of Masson’s Trichrome staining revealed significant differences in collagen deposition among the groups by postoperative day 13 (Fig. 6). Collagen deposition, expressed as the collagen area fraction (percentage of blue-stained area relative to the total tissue area), was 49.43 ± 0.41% in the control group, which was significantly lower than that observed in the HCS group (61.01 ± 0.75%) and the PHCS group (88.76 ± 0.35%) (HCS vs. control, *p* < 0.05; PHCS vs. control, *p* < 0.05; PHCS vs. HCS, *p* < 0.05).

## Discussion

The hBMSCs, known for their multipotent differentiation capacity, are promising candidates for tissue engineering applications [23]. Although conventional mesenchymal cell sheets (MCSs) provide abundant cellular and extracellular matrix components, their clinical application is limited by mechanical fragility and insufficient vascularization [24]. To overcome these limitations, we developed a PHCS strategy, which involves co-culturing hBMSCs with HUVECs under hypoxic conditions (2% O_2_) within a 3D scaffold. This approach promotes the formation of capillary-like networks through multiple mechanisms: (1) pericytic differentiation of hBMSCs to stabilize nascent vessels [25], (2) enhanced secretion of angiogenic factors such as VEGF and TGF-β1 [19, 26], and (3) Notch signaling activation mediated by direct cell-cell contact [27].

Compared to non-vascularized HCS grafts, PHCS significantly enhanced wound healing outcomes. These findings suggest that PHCSs improve oral mucosal wound healing via multiple angiogenesis-related mechanisms. Notably, PHCS treated grafts achieved complete wound closure by day 10, earlier than the HCS and control groups (day 13), indicating a shortened inflammatory phase (*p* < 0.05), consistent with prior findings that prevascularization enhances graft survival through early neovascularization [28].

Histologically, PHCS grafts demonstrated reduced neutrophil infiltration and enhanced glandular regeneration (Fig. 4), supporting the emerging concept that vascular architecture can modulate the immune microenvironment [29]. Compared with HCS, the PHCS group exhibited a 2.1-fold increase in mature glandular structures and an obvious increase in collagen deposition (*p* < 0.05), likely due to improved oxygen and nutrient delivery, as supported by recent perfusion studies [30]. Interestingly, the vascular profile in PHCS grafts featured fewer but larger functional vessels (Fig. 5), indicative of advanced vascular remodeling [31].

Epithelial thickness dynamics (Fig. 3) further illustrated the distinct healing patterns among groups. On postoperative day 3, the HCS group showed reduced epithelial thickness compared to controls; by day 7, thickness between the two groups had converged. This may be attributed to paracrine factors (e.g., VEGF, bFGF, TGF-β1) secreted by hBMSCs in HCS, promoting early epithelial remodeling, reducing inflammatory edema, and facilitating epithelial cell migration rather than proliferation [19, 32, 33]. Additionally, the immunomodulatory properties of hBMSCs may suppress TNF-α and IL-1β expression [34, 35], while the cell sheet structure acts as a barrier to limit inflammatory infiltration and granulation overgrowth [36, 37]. By day 7, as the control group entered the proliferative phase, epithelial thickness aligned with HCS, although the latter exhibited a more organized and differentiated epithelial architecture [38].

Throughout the healing process, the PHCS group demonstrated superior epithelial regeneration. By day 3, epithelial thickness was similar to controls, but with markedly higher cell density and stratification, indicating early activation of the repair process. Prevascularized structures in PHCS enhanced oxygen and nutrient supply, stimulated orderly epithelial proliferation, and activated basal stem cells via bFGF and VEGF signaling [39], promoting early stratification. Although keratinization remained incomplete at this stage, the proliferative activity was evident. By day 7, PHCS sustained growth factor release through stable microcirculation [40], accelerating epithelial cell proliferation, migration, and differentiation, resulting in significantly increased epithelial thickness. Enhanced fibroblast activity and mesenchymal-epithelial interactions further promoted tissue reconstruction [41]. By day 13, the PHCS and HCS groups had comparable epithelial thickness, but the PHCS epithelium exhibited higher maturity and density, whereas the control group lagged behind.

Collagen remodeling analysis (Fig. 6) further highlighted the capacity of PHCS to accelerate extracellular matrix maturation. By postoperative day 13, PHCS grafts exhibited a significantly greater collagen deposition area compared with the control group (*p* < 0.05), as determined by semi-quantitative analysis of Masson’s Trichrome-stained sections. Histologically, the collagen-rich regions in PHCS-treated wounds appeared denser and more uniformly distributed within the lamina propria. It should be emphasized that collagen evaluation in this study was based on the relative area of collagen deposition rather than quantitative assessment of fiber alignment or orientation. Nevertheless, the observed histological features are consistent with previous reports indicating that enhanced vascular support improves matrix deposition and maturation during wound healing [42]. Given that oral mucosa is continuously subjected to mechanical forces such as compression, tension, and shear, timely collagen accumulation within the connective tissue is essential for restoring tissue integrity and function [43, 44]. The transition from loosely distributed collagen to more compact collagen-rich regions in the PHCS group suggests accelerated progression through the remodeling phase, which may contribute to improved biomechanical stability and reduced scar formation potential [45].

Although the total microvessel number in PHCS grafts was lower, the cross-sectional area per vessel was significantly greater, suggesting a more mature and efficient angiogenic process. This phenomenon can be explained by several mechanisms: (1) prevascular structures in PHCS may have progressed to later stages of vascular development, characterized by vessel fusion and lumen expansion, resulting in fewer but more stable and perfusion-efficient vessels; (2) enhanced pericyte recruitment (e.g., α-SMA-positive cells) further stabilized vasculature, consistent with vascular pruning processes [46–48]; (3) improved local hemodynamics, such as increased shear stress, may have driven the restructuring of the vascular network toward fewer, larger vessels for optimized perfusion [49]; and (4) accelerated wound healing in the PHCS group likely promoted earlier entry into the tissue remodeling phase, during which angiogenesis slows and vascular networks become more organized [50]. Additionally, embedded vascularized cell sheets may secrete angiopoietin-1, PDGF-BB, and other regulatory factors to maintain long-term vascular stability and maturation [46, 51, 52].

This study demonstrates the regenerative benefits of PHCS in oral mucosal healing based on histological evidence, but several limitations remain. First, collagen assessment was performed using a semi-quantitative area-based method derived from Masson’s Trichrome staining, which reflects the extent of collagen deposition rather than fiber alignment, orientation, or ultrastructural organization. More advanced techniques, such as collagen alignment index analysis, polarized light microscopy, or second harmonic generation imaging, may provide additional insights into collagen fiber architecture and maturation in future studies. Second, although immunocompetent animals allowed short-term graft survival and vascularization, which is consistent with previous studies [19], the use of immunodeficient models in future work will help minimize immune-related confounders and strengthen evidence. Third, while histology implied possible involvement of VEGF and TGF-β1, these factors were not directly measured. Subsequent studies will apply ELISA or immunohistochemistry to quantify protein expression and clarify molecular mechanisms. Further multi-omics and in vitro analyses are needed to fully elucidate the cellular and regulatory pathways underlying PHCS-mediated repair.

## Conclusion

Overall, this study provides histological evidence supporting the regenerative advantages of PHCS in oral mucosal healing. PHCS significantly enhances oral mucosal wound healing through multiple synergistic mechanisms, including robust angiogenesis, accelerated epithelial regeneration, improved collagen deposition, and immunomodulation. Key findings indicate that PHCS grafts promote rapid and functional tissue repair, as evidenced by earlier wound closure, enhanced glandular regeneration, increased collagen deposition, histologically advanced matrix remodeling, and the formation of stable, perfusable vascular networks.

## Data Availability

The datasets used and/or analysed during the current study are available from the corresponding author on reasonable request.

## Abbreviations

STSGs: Split-thickness skin grafts
hBMSC: Human bone marrow-derived mesenchymal stem cell
HCS: Human bone marrow-derived mesenchymal stem cell sheets
PHCS: Pre-vascularized human bone marrow-derived mesenchymal stem cell sheets
HUVECs: Human umbilical vein endothelial cells
3D: Three-dimensional
α-MEM: α-minimum essential medium
HE: Hematoxylin and eosin (HE) staining
HPF: High-power field
MCSs: Mesenchymal cell sheets
